# Inhibition of HDAC Enzymes Contributes to Differential Expression of Pro-Inflammatory Proteins in the TLR-4 Signaling Cascade

**DOI:** 10.3390/ijms21238943

**Published:** 2020-11-25

**Authors:** Ulrike Weiss, Moritz Möller, Sayed Adham Husseini, Christine Manderscheid, Julia Häusler, Gerd Geisslinger, Ellen Niederberger

**Affiliations:** 1 Pharmazentrum frankfurt/ZAFES, Institute of Clinical Pharmacology, Faculty of Medicine, Goethe-University Frankfurt, Theodor Stern Kai 7, 60590 Frankfurt am Main, Germany; weiss@med.uni-frankfurt.de (U.W.); m_moeller@mail.de (M.M.); s6997994@stud.uni-frankfurt.de (S.A.H.); Manderscheid@med.uni-frankfurt.de (C.M.); JuliaHa@web.de (J.H.); geisslinger@em.uni-frankfurt.de (G.G.); 2 Fraunhofer Institute for Molecular Biology and Applied Ecology, Branch Translational Medicine (IME-TMP) and Fraunhofer Cluster of Excellence for Immune mediated diseases (CIMD), Theodor Stern Kai 7, 60590 Frankfurt am Main, Germany

**Keywords:** HDAC, acetylation, macrophages, inflammation

## Abstract

Class I and II histone deacetylases (HDAC) are considered important regulators of immunity and inflammation. Modulation of HDAC expression and activity is associated with altered inflammatory responses but reports are controversial and the specific impact of single HDACs is not clear. We examined class I and II HDACs in TLR-4 signaling pathways in murine macrophages with a focus on IκB kinase epsilon (IKKε) which has not been investigated in this context before. Therefore, we applied the pan-HDAC inhibitors (HDACi) trichostatin A (TSA) and suberoylanilide hydroxamic acid (SAHA) as well as HDAC-specific siRNA. Administration of HDACi reduced HDAC activity and decreased expression of IKKε although its acetylation was increased. Other pro-inflammatory genes (IL-1β, iNOS, TNFα) also decreased while COX-2 expression increased. HDAC 2, 3 and 4, respectively, might be involved in IKKε and iNOS downregulation with potential participation of NF-κB transcription factor inhibition. Suppression of HDAC 1–3, activation of NF-κB and RNA stabilization mechanisms might contribute to increased COX-2 expression. In conclusion, our results indicate that TSA and SAHA exert a number of histone- and HDAC-independent functions. Furthermore, the data show that different HDAC enzymes fulfill different functions in macrophages and might lead to both pro- and anti-inflammatory effects which have to be considered in therapeutic approaches.

## 1. Introduction

Histone proteins as carriers of DNA in eukaryotes are able to influence gene expression depending on several post-translational histone modifications such as phosphorylation, methylation and acetylation [1,2,3]. Changes in histone acetylation are tightly regulated by histone acetyltransferases (HATs) and histone-deacetylases (HDACs). Histone acetylation by HATs leads to relaxation of the chromatin structure which allows binding of transcription factors followed by increased gene expression. Consequently, deacetylation by HDACs tightens binding of DNA to the histones and hinders transcription factor activity [4]. In addition to the deacetylation of histones, it is clear that HDACs also target other cytoplasmic proteins [5,6]. HDACs contribute to innate immunity by regulation of Toll-like receptor (TLR) and interferon (IFN) signaling [7], and adaptive immunity by modulation of antigen presentation and B- and T-cell function [8]. So far, 18 mammalian HDAC genes have been identified, which are grouped into four classes comprising class I (HDAC 1, 2, 3, 8) and class II (HDAC 4, 5, 6, 7, 9, 10) as classical HDACs, the sirtuins as class III and HDAC 11 as class IV [7,9]. Class I HDACs are mainly involved in innate immunity by regulation of inflammatory reactions while class II HDACs are associated with adaptive immunity [10]. Macrophages play a central role in innate immunity and inflammatory responses by driving initiation as well as resolution of inflammation. Upon inflammatory stimulation e.g., with lipopolysaccharide (LPS), IL-1β or IFNγ they release cytokines and express a number of pro- and anti-inflammatory genes dependent on transcriptional, post-transcriptional and epigenetic mechanisms. Histone modification is discussed as one major epigenetic modulation in the process of macrophage activation and polarization, but the specific role of histone acetylation in the context of inflammation and inflammatory macrophages is still far from being completely elucidated [11]. In this study, we intended to gain more insight into this topic using the pan-HDAC inhibitors trichostatin A (TSA) and suberoylanilide hydroxamic acid (SAHA) as well as different HDAC-specific siRNAs in RAW264.7 murine macrophages. TSA and SAHA are both nonselective small molecule inhibitors of HDACs which are considered as suitable therapeutics for various malignant diseases. SAHA, also known as Vorinostat, is approved in some countries for the treatment of cutaneous T-cell lymphoma and in clinical testing for other cancer types and HIV. Furthermore, these compounds are discussed increasingly for their potential use in inflammatory diseases [12,13]. Modulation of NF-κB, an important transcription factor in inflammatory processes, as well as its classical activating inhibitor κB-kinase (IKK) complex have already been associated with inhibition of HDAC activity [14,15]. IKKε however, an alternative NF-κB activating kinase also known for its role in inflammatory processes [16,17,18], has not been linked with histone acetylation so far. We investigated the impact of the HDACi on inflammatory gene expression with a focus on IKKε and additionally used siRNAs against different HDACs to figure out which specific HDAC is involved in the differential target regulations.

## 2. Results

### 2.1. Effects of HDAC Inhibitors on Cell Proliferation and Apoptosis Induction

**Figure 1:**
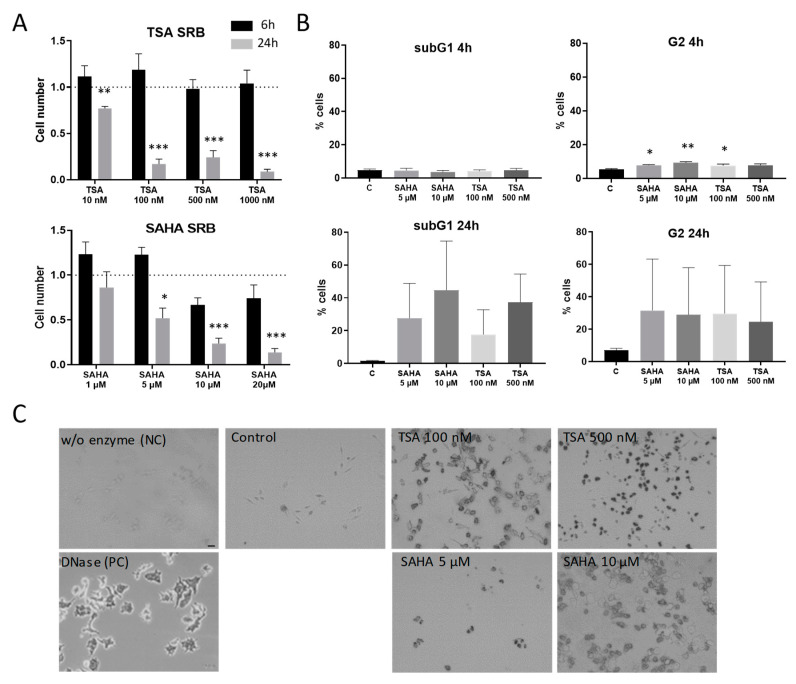
Effects of trichostatin A (TSA) and suberoylanilide hydroxamic acid (SAHA) on cell proliferation and apoptosis induction. (**A**): Cytotoxicity and cell proliferation were assessed by the sulforhodamine B (SRB) assay after 6 and 24 h incubation time, respectively, at different concentrations of TSA and SAHA (6 h: n = 4; 24 h: n = 3). The cell number of vehicle-treated cells was set as “1” indicated by the dotted line. (**B**): Flow cytometric analysis of cell cycle distribution and apoptosis induction 4 and 24 h after treatment with TSA and SAHA (n = 4). (**C**): TUNEL staining 24 h after TSA and SAHA administration. A preparation without enzyme served as negative control, cells treated with DNase as positive control. The picture shows representative stainings from three independent incubations. Scale Bar: 50 µm. * *p* < 0.05, ** *p* < 0.01, *** *p* < 0.001; statistically significant difference as compared to vehicle-treated control.

Since it is already well-known that HDAC inhibitors induce apoptosis in a variety of cells, it was important to evaluate concentrations and incubation times that avoid effects that occur mainly due to apoptotic processes. Therefore, RAW264.7 murine macrophages were subjected to cell proliferation and cytotoxicity assays. After 6 h treatment, TSA and SAHA did not significantly reduce proliferation in macrophages. In contrast, 24 h incubation with TSA and SAHA led to a significant concentration-dependent reduction in the cell number (Figure 1A). To assess if the decrease in living cells is due to effects on cell cycle and apoptosis, we additionally performed FACS and TUNEL assays. FACS analysis supported the results from the cell proliferation assays and showed no induction of cell death after 4 h. However, a slight-but-significant arrest in the G2 phase of the cell cycle was already detectable for both TSA and SAHA. After 24 h, there was a massive increase of cells in the G2 phase and the subG1 fraction indicating induction of apoptosis (Figure 1B) which was further supported by TUNEL staining (Figure 1C). These data prompted us to perform most experiments in the inflammatory context with incubation times of 4 and 6 h and concentrations of 100 and 500 nM TSA and 5 and 10 µM SAHA. These concentrations are relatively high considering the IC_50_ values for HDAC inhibition of TSA and SAHA in cell-free assays. However, the concentration range is in line with other studies which investigated TSA and SAHA in cell culture experiments [19,20,21].

### 2.2. Impact of TSA and SAHA on the Expression of Pro-Inflammatory Genes

**Figure 2:**
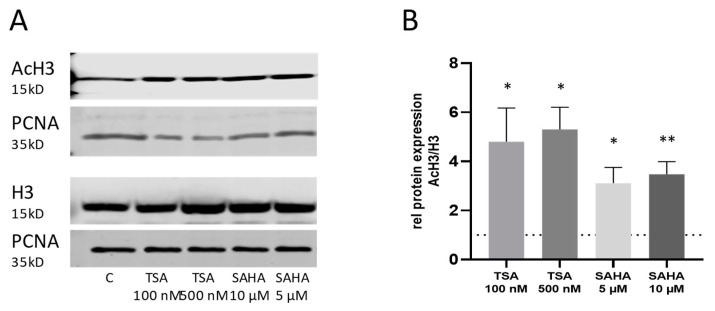
Histone-deacetylase (HDAC) activity after TSA and SAHA treatment. (**A**): Western blot analysis of nuclear histone H3 acetylation after 4 h incubation with TSA and SAHA at the indicated concentrations. The pictures show representative blots. (**B**): Densitometric analysis of all blots (n = 4–5/group). For better comparison, the acetylation levels were normalized to vehicle-treated control cells which have been set as “1” indicated by the dotted line. * *p* < 0.05, ** *p* < 0.01 statistically significant difference as compared to vehicle-treated control. AcH3: Acetyl histone H3; PCNA: proliferating-cell-nuclear-antigen.

The efficacy of TSA and SAHA to inhibit HDAC activity was proven by a significant increase in acetylated histone H3 protein (Figure 2).

**Figure 3:**
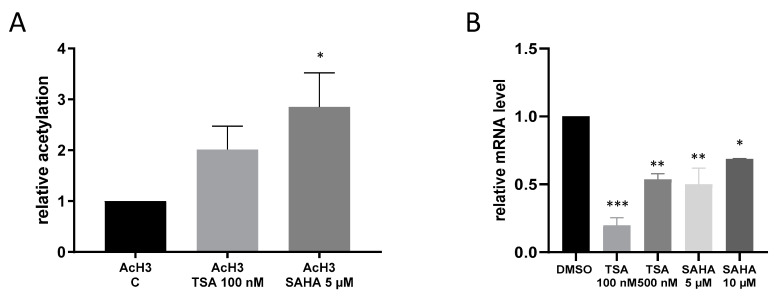
Acetylation of the IκB kinase epsilon (IKKε) promoter. (**A**): RAW264.7 cells were incubated for 16 h with TSA and SAHA. ChIP analysis of nuclear extracts was carried out with antibodies against acetylated histone H3 and histone H3 followed by qRT-PCR analysis using primers against the IKKε promoter sequence. (n = 6), (**B**): IKKε mRNA expression after treatment with TSA and SAHA (n = 3), * *p* < 0.05, ** *p* < 0.01, *** *p* < 0.001 statistically significant difference as compared to vehicle control.

Since IKKε has so far never been linked with acetylation mechanisms, we performed ChIP analysis to assess whether or not HDAC inhibition by TSA and SAHA is at all able to affect acetylation of the IKKε promoter. The results show that TSA and SAHA induced an increase in IKKε promoter acetylation. However, IKKε mRNA expression was significantly downregulated in macrophages by treatment with HDACi which does not correspond with the conventional view that augmented acetylation leads to a rise in gene expression (Figure 3). 

**Figure 4:**
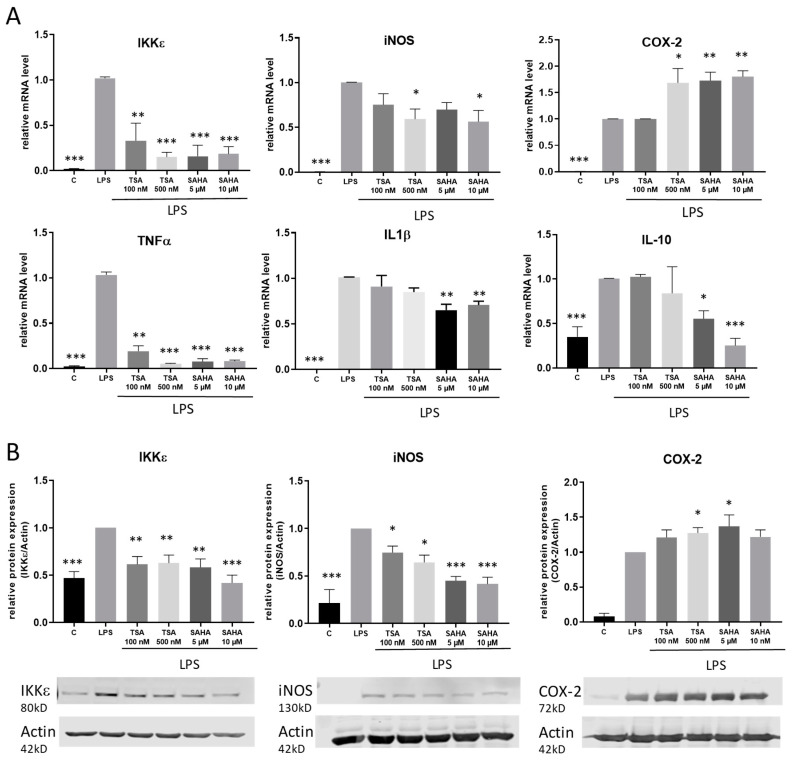
Effects of HDACi on lipopolysaccharide (LPS)-induced inflammatory gene expression. (**A**): mRNA expression analysis of inflammatory genes 4 h after LPS incubation with and without addition of TSA and SAHA at the indicated concentrations. (**B**): Western blot analysis of inflammatory genes after treatment of cells with LPS with and without SAHA or TSA for 6 h, respectively. The blots show one representative result, the diagrams the densitometric analysis of all blots (n = 5–6), * *p* < 0.05, ** *p* < 0.01, *** *p* < 0.001, statistically significant difference as compared to LPS-treated control.

To assess the impact of the HDACi in a pro-inflammatory setting, macrophages were stimulated with the TLR-4 agonist LPS. The regulation of IκB kinase epsilon (IKKε) as well as various further pro-inflammatory genes and cytokines including cyclooxygenase 2 (COX-2), inducible nitric oxide synthase (iNOS), interleukin-1 (IL-1) beta and tumor necrosis factor (TNF) alpha was analyzed by qRT-PCR and western blot. After 4 h incubation time, we found different regulations of the distinct inflammatory genes. Similar to treatment without LPS, TSA and SAHA both significantly inhibited the LPS-induced expression of IKKε. Furthermore, iNOS and TNFα were significantly downregulated by HDACi. TSA had no effect on IL-1β while SAHA reduced its expression. LPS-induced COX-2 expression was further increased by TSA and SAHA in a significant manner. In addition, interleukin 10 (IL-10) was investigated as a potent proresolving gene. IL-10 expression was enhanced by LPS and this effect was not altered after treatment with TSA but significantly decreased by SAHA (Figure 4A). The results were confirmed on the protein level by showing that LPS-induced IKKε and iNOS protein expression was inhibited by TSA and SAHA while COX-2 was significantly increased by both drugs (Figure 4B). After evaluating that IKKε is downregulated by HDACi, we additionally wanted to know if a regulation of IKKε might have an impact on HDACi-induced modulations of gene expression. Therefore, we prepared IKKε wildtype and knock-out bone-marrow-derived macrophages and performed exemplary expression analysis of iNOS and TNFα. The results show no difference between the genotypes indicating that HDACi-induced effects are not mediated via IKKε (Figure S1).

### 2.3. TSA and SAHA Enhance LPS-Induced NF-κB Activation

**Figure 5:**
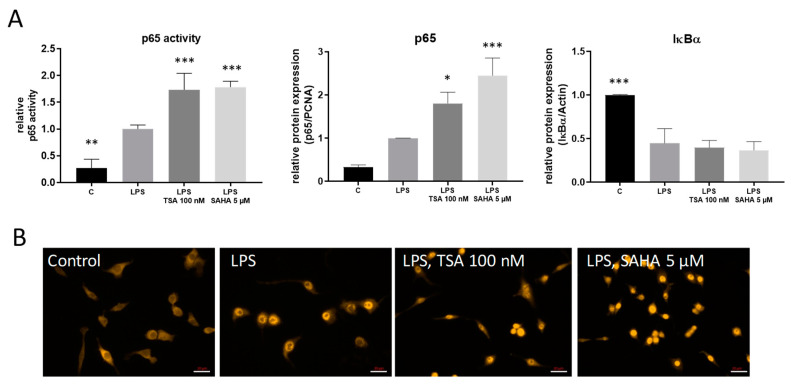
Effects of HDAC inhibition on LPS-induced NF-κB transcription factor activity. (**A**): p65 transcription factor analysis as assessed by transcription factor ELISA (TransAM, left panel), nuclear p65 translocation (middle panel) and cytosolic I-κBa degradation (right panel) by western blot analysis (n = 6). The diagrams show the densitometric analysis of all blots. (**B**): Immunofluorescence staining of RAW264.7 cells after vehicle treatment or incubation with LPS for 30 min with and without addition of TSA and SAHA. The pictures show representative results from three independent incubations. Scale Bar: 20 µm. * *p* < 0.05, ** *p* < 0.01, *** *p* < 0.001, statistically significant difference as compared to LPS-treated control.

The transcription factor NF-κB is involved in the regulation of several pro-inflammatory genes after TLR-4 stimulation. Therefore, regulation of these genes also might be influenced via HDACi-induced changes in NF-κB activation. LPS treatment reduced the expression of the inhibitor of kappa B protein alpha (IκBα), thereby allowing the release of NF-κB from the trapping complex with IκBα and its translocation into the nucleus where it binds to the DNA of its target genes. Unexpectedly, treatment with TSA and SAHA augmented these effects as assessed by transcription factor ELISA, western blot and immunofluorescence (Figure 5A,B). These results indicate that the HDACi do not inhibit NF-κB but rather enhance its activation which might contribute to the increase in COX-2 levels. 

### 2.4. Effects of TSA and SAHA Are Only Partially Mediated by Histone Modulations

**Figure 6:**
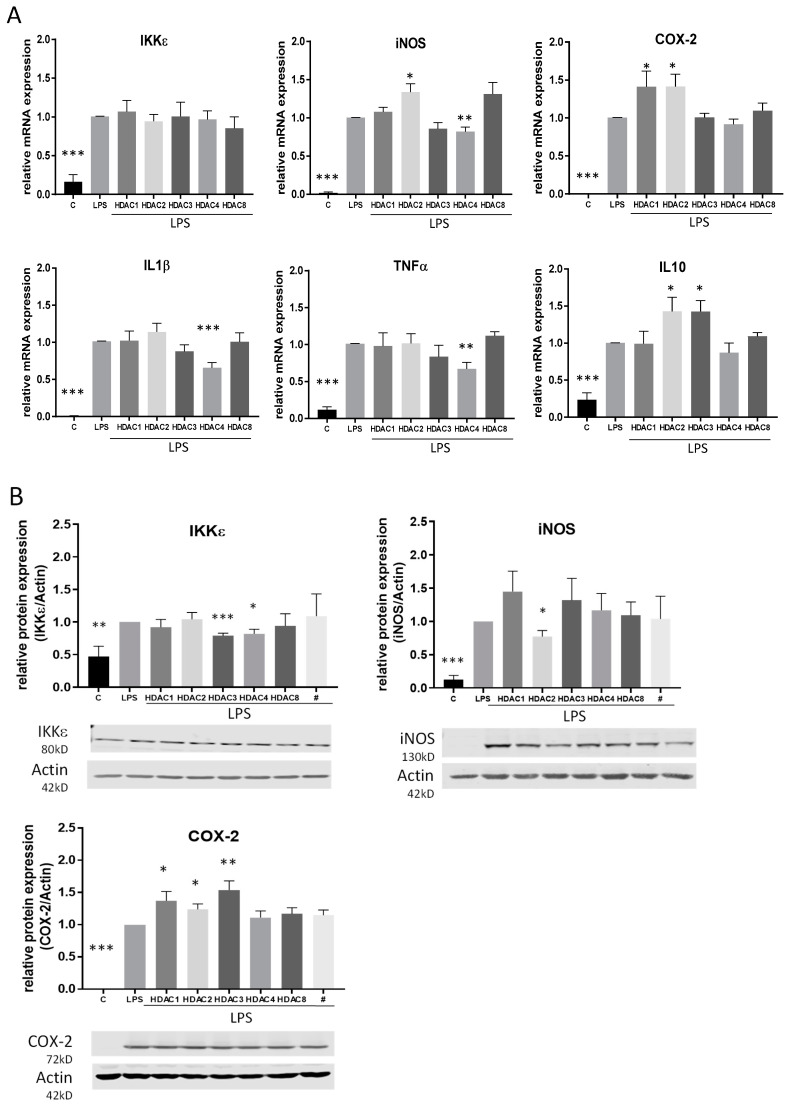

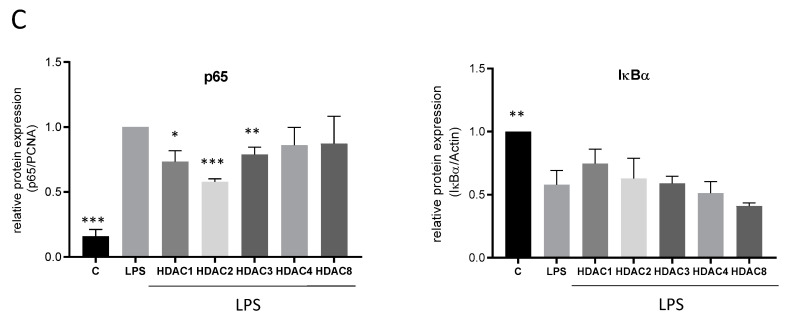
Analysis of LPS-induced inflammatory genes and NF-κB transcription factor activity after knock-down of HDACs by siRNA. (**A**): mRNA expression analysis of inflammatory genes after LPS incubation of RAW264.7 cells with and without knock-down of different HDAC enzymes (n = 5–6), (**B**): Western blot analysis of inflammatory genes after treatment of control and HDAC knock-down cells with LPS. The blots show one representative result, the diagrams the densitometric analysis of all blots (n = 5–6). (**C**): Densitometric analysis of western blot analyses for p65 nuclear translocation and cytosolic I-κBa degradation after treatment of RAW264.7 cells with HDAC siRNAs and LPS. Actin served as loading control for cytosolic extracts, PCNA as control for nuclear extracts, (n = 4), * *p* < 0.05, ** *p* < 0.01, *** *p* < 0.001, statistically significant difference as compared to LPS-treated control.

To gain a better overview of the HDACs which are responsible for the HDACi-mediated effects, we performed further experiments using siRNA directed against HDAC 1, 2, 3, 4 and 8. qRT-PCR analyses confirmed that all siRNAs are able to downregulate their respective target HDAC. Furthermore, acetylation of histone H3 was increased after treatment with HDAC siRNA. We also assessed the impact of the siRNAs on cell proliferation and found that only HDAC 3 and 4 siRNA showed a slight but significant reduction of cell proliferation (Figure S2). Interestingly, gene expression analysis after siRNA transfection was only partially consistent with the results for HDACi treatment and indicated that different HDACs are involved in the different gene modulations. At mRNA level, IL-1β, TNFα and iNOS were significantly downregulated by siRNA against HDAC 4 and COX-2 was significantly upregulated after treatment with HDAC 1 and 2 siRNA. In contrast to the results with TSA and SAHA, iNOS and IL-10 were upregulated after HDAC 2 siRNA and IL-10 also after HDAC 3 siRNA treatment. siRNA against HDAC 8 did not affect any gene. None of the siRNAs had an impact on IKKε mRNA (Figure 6A). On the protein level, COX-2 was again upregulated after HDAC 1 and 2 and additionally after HDAC 3 siRNA. In contrast to mRNA regulation, the iNOS protein level was decreased after HDAC 2 siRNA application and the IKKε protein level was downregulated after siRNA against HDAC 3 and 4 (Figure 6B).

Interestingly, while TSA and SAHA induced an upregulation of NF-κB, HDAC 1, 2 and 3 siRNAs significantly reduced NF-κB activity (Figure 6C) supporting the assumption that several TSA- and SAHA-induced effects are mediated independently of HDAC and histone acetylation, respectively.

**Figure 7:**
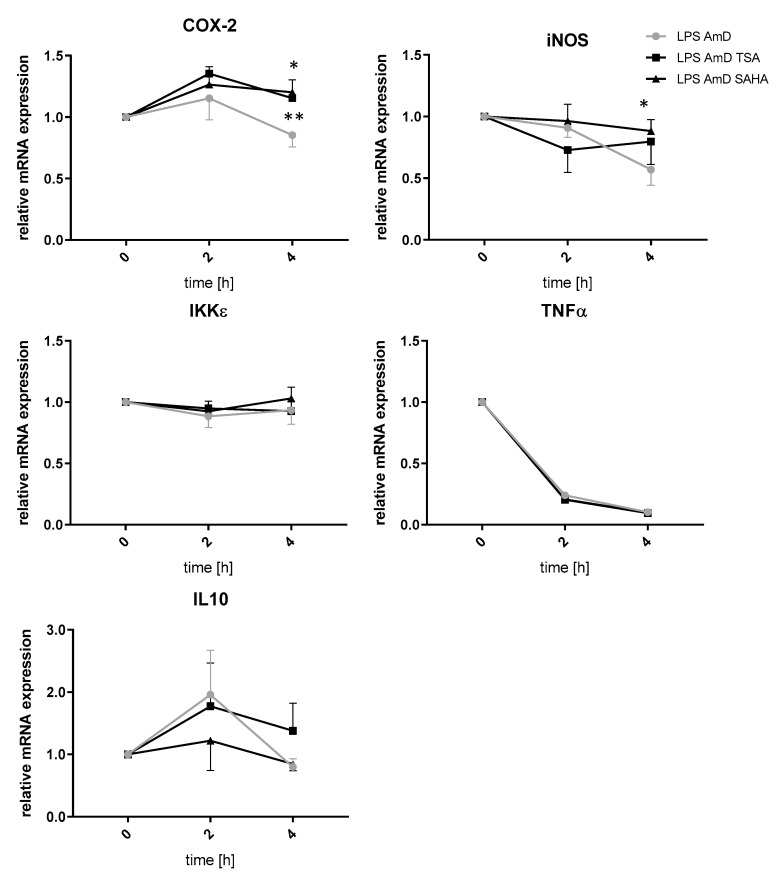
Effects of TSA and SAHA on mRNA stability of LPS-induced inflammatory genes. mRNA stability was assessed by blocking transcription with actinomycin (AmD) (1 µg/mL). RAW264.7 cells were incubated with LPS for 24 h before TSA and SAHA were added together with actinomycin for 2 and 4 h. The time point of actinomycin addition was set as baseline (“1”). (n = 4). * *p* < 0.05, ** *p* < 0.01, statistically significant difference as compared to LPS/actinomycin-treated control.

Since a decrease in gene expression after inhibition of HDAC might be rather an indirect effect of HDACi or siRNA, respectively, we prompted it might be due to increased mRNA degradation, a mechanism which has already been described after HDAC inhibition. To investigate this topic, we performed experiments with actinomycin which blocks transcriptional activity. The analyses indicated that increased mRNA degradation is not involved in the downregulation of IKKε, TNFα, iNOS and IL-10 after TSA and SAHA treatment. In contrast, iNOS and COX-2 mRNA were significantly stabilized after 4 h incubation with TSA and SAHA, respectively (Figure 7).

## 3. Discussion

HDACs have been described as positive and negative regulators of TLR signaling. Accordingly, HDACi are associated with inhibiting as well as enhancing effects on the expression of pro-inflammatory genes [5,19,20]. However, the exact mechanisms including regulation of HDACs by inflammatory conditions and specific HDACs involved are not completely clarified yet. Furthermore, the impact of the noncanonical IκB Kinase epsilon which has been repeatedly associated with inflammatory processes and inflammatory diseases [16,17,18] is not known in this context so far. Current evidence suggests that class I HDACs are the main players in innate immunity by regulation of inflammatory reactions while class II HDACs are mostly involved in mechanisms of adaptive immunity [10]. This study focused on four class I HDACs (1, 2, 3, 8) and one class II HDAC (4) and their potential impact on LPS-induced TLR-4 signaling in murine macrophages. To modulate HDAC function, the pan-HDAC inhibitors TSA and SAHA, as well as specific siRNAs for the respective HDACs, have been applied. Since both small molecule HDACi have been described for their cytotoxic effects [22], we first evaluated their influence on cell proliferation and apoptosis induction and then adjusted experimental conditions to exclude effects relying on apoptosis induction.

In accordance with contrary effects on pro-inflammatory genes described for HDAC inhibitors [19,20], our data showed different groups of potential effector proteins after treatment of macrophages with TSA and SAHA. While LPS-mediated induction of the pro-inflammatory genes IKKε, TNFα, IL-1β and iNOS was significantly inhibited, COX-2 was significantly enhanced. iNOS and TNFα downregulations have already been described after knock-down of class I HDAC, particularly 1, 2 and 3, and after treatment of macrophages with TSA, respectively [23,24,25,26,27,28]. Our siRNA data suggest that the decrease in iNOS protein expression is dependent on inhibition of HDAC 2 while TNFα reduction might be due to the knock-down of HDAC 4. To the best of our knowledge, there is no report on effects of HDACi on IKKε so far. IKKε is an activator of IRF as well as NF-κB pathways [29] which was downregulated in our study after TSA- and SAHA-treatment as well as after administration of siRNA against HDAC 3 and 4. This fits well with studies showing that TLR-induced activation of target genes in the IRF family is positively regulated by HDAC in macrophages and dendritic cells [7]. However, the mechanism behind the IKKε downregulation is not completely clear. Increased acetylation of the IKKε promoter was detectable and corresponds with inhibition of deacetylase activity but cannot be attributed directly to decreased protein regulation. Therefore, an indirect effect has to be assumed as, for instance, in case of treatment with siRNA against HDAC 3 which decreased NF-κB activation potentially leading to IKKε downregulation.

In contrast to downregulated genes after HDACi treatment, the LPS-induced increase in COX-2 mRNA and protein expression was further augmented by TSA and SAHA. This is in accordance with previous studies showing that increased acetylation of the COX-2 promoter by overexpression of HAT or inhibition of HDAC was associated with an increased expression of COX-2 [19,20,30,31]. It has been suggested that this effect is HDAC-specific and mediated by distinct HDACs. HDAC 1, as well as HDAC 8 overexpression, was associated with inhibition of LPS-induced COX-2 upregulation [19,30]. Administration of MS-275, a drug that preferentially inhibits HDAC 1, increased COX-2 mRNA level in primary macrophages, however only at concentrations which are cytotoxic [20]. In our study, HDACs 1, 2 and 3 were identified as negative regulators of COX-2 expression thus further supporting the assumption of direct and HDAC-specific effects.

Direct and indirect effects of HDAC inhibition have already been described and it is well-known that several other proteins apart from histones are acetylated and regulated by this means [6,32]. In particular, negative regulations of LPS-induced inflammatory genes by HDACi are most likely indirect effects. Several studies revealed that HDACi may also act as regulators of the transcription factors PU.1, C/EBP and c-jun and thereby contribute to protein modulations [19,26,31]. NF-κB is another important transcription factor in the regulation of TLR-4-induced pro-inflammatory genes. Its activation is adjusted by a number of post-translational mechanisms including acetylation. In addition, PU.1 is also involved in its activation mechanism [33,34]. Previous results on p65 regulation after HDAC inhibition are conflicting which is probably due to the fact that activation of NF-κB can either be enhanced or inhibited respective to the site of acetylation [35,36]. Some reports show no regulation of p65 expression and activity after knock-down of HDAC or inhibition with TSA, respectively [31,37,38]. Others reveal an inhibition but mostly after long incubation times or high concentrations [25,39,40] and further studies indicate that class I HDACs negatively regulate NF-κB by deacetylation of the p65 subunit [7,41]. We observed an enhanced NF-κB p65 activity after treatment with TSA and SAHA which might at least partially contribute to increased COX-2 expression but not to downregulation of other pro-inflammatory genes. In contrast, specific downregulation of distinct HDACs led to NF-κB inhibition which might then participate in the reduced expression of IKKε and iNOS. These data support the assumption that different HDACs might act at different acetylation sites of their target genes and that TSA and SAHA might additionally exert completely HDAC-independent effects.

In conclusion, our data confirm that small molecule HDAC inhibitors show a number of effects on inflammatory genes which can be only partially attributed directly to histone acetylation. Furthermore, the results support the hypothesis that differential HDACs exert divergent functions on inflammatory genes. These varying effects, as well as potential histone-independent properties of HDAC inhibitors, are important considering their potential use as anticancer or anti-inflammatory drugs. In the case of COX-2, upregulation might be problematic in the context of inflammatory diseases while downregulation of other genes will particularly contribute to beneficial effects. So far, it is difficult to predict the unwanted side effects of such drugs indicating that further research is necessary to clarify the medical indications where HDACi may act as suitable drugs.

## 4. Materials and Methods

### 4.1. Drugs

Trichostatin A (TSA) and suberoylanilide hydroxamic acid (SAHA, also known as Vorinostat), used as panHDAC inhibitors, were purchased from TOCRIS (Darmstadt, Germany) and dissolved in dimethyl sulfoxide (DMSO) (Sigma, Deisenhofen, Germany) in a stock solution of 100 mM. Dilutions were performed with DMSO to adjust the DMSO concentration in each incubation to 0.1%. siRNAs against the respective HDAC as well as negative control oligonucleotides were purchased from Thermo Fisher Scientific (Schwerte, Germany). Lipopolysaccharide (LPS) was purchased from Sigma (Darmstadt, Germany).

### 4.2. Cells

Murine RAW264.7 macrophages were purchased from LGC Standards (Wesel, Germany). Cells were cultured and incubated in advanced Dulbecco’s modified eagle medium (DMEM) containing 10% fetal calf serum (FCS) (Thermo Fisher Scientific, Schwerte, Germany). Incubation with TSA and SAHA was performed for the indicated times and concentrations. Since the drugs were dissolved in DMSO, solvent concentrations were adjusted to 0.1% in all incubations and 0.1% DMSO alone was used as vehicle control. For initial analysis of cytotoxic effects of the drugs, cells were incubated with TSA and SAHA for 6 and 24 h to determine a useful time frame for further incubations.

Bone-marrow-derived macrophages (BMM) were generated from IKKε^−/−^ and wildtype mice. Therefore, the hind legs were cut directly beyond and above the joints. The bones were cleaned from muscle and connective tissues and then placed into a perforated 0.2 mL tube, which was inserted into a 1.5 mL reaction tube. The tubes were centrifuged at 16,800× *g* for 15 s, and bone marrow cell containing pellet was resuspended in RPMI 1640 medium containing 10% FCS, 1% Penicillin/Streptomycin (Thermo Fisher Scientific, Schwerte, Germany) and 20 ng/mL recombinant murine macrophage colony stimulating factor (M-CSF, Peprotech, Hamburg, Germany). Subsequently, cells were plated in 6-well plates and cultivated and differentiated for 7 d before stimulation.

Stimulation of the cells with LPS was performed simultaneously with TSA and SAHA for the determination of gene expression with western blot and qRT-PCR. Due to different periods needed for mRNA transcription and protein translation, cells were incubated for 4 h for mRNA analysis while protein expression was determined after 6 h of incubation time. For RNA stability, we prestimulated the expression of inflammatory genes for 24 h and then added actinomycin (1 µg/mL) (Sigma, Deisenhofen, Germany) with and without TSA and SAHA for 2 and 4 h before mRNA preparation took place.

### 4.3. siRNA

RAW264.7 cells were seeded on 24-well plates at a density of 2 × 10^5^ cells/well and cultivated for 24 h. Pre-evaluated siRNAs against HDAC 1, 2, 3, 4 and 8 as well as scrambled control oligonucleotides were purchased from Thermo Fisher Scientific (Schwerte, Germany) (Silencer Select siRNA against mouse HDAC). Transfection of RAW264.7 cells was performed using Lipofectamine RNAiMAX transfection reagent (Thermo Fisher Scientific (Schwerte, Germany) as recommended by the manufacturer. Briefly, siRNA as well as Lipofectamine were mixed with Opti-MEM medium in separate tubes and were combined and incubated for 5 min at room temperature. Then, the lipofectamine—siRNA mix was added to the cell culture medium. Cells were incubated with siRNA over a time period of 42 h before incubation with LPS started. For siRNA experiments, Lipofectamine-treated macrophages were used as controls.

### 4.4. Sulforhodamine B Cytotoxicity Assay

The sulforhodamine B (SRB) assay has been used to determine cell density, based on the analysis of the cellular protein content [42]. For the sulforhodamine B assay, cells were seeded in quadruplicates in 24-well tissue culture plates (2 × 10^4^ cells/well) and cultured for 6 and 24 h. At the end of the cultivation period, the supernatant was discarded and cells were fixed with 5% trichloroacetic acid (TCA) for 1 h at 4 °C. The plates were washed seven times with H_2_O and then dried for 1 h at 60 °C. Staining of cellular proteins was performed for 30 min at RT with sulforhodamine B (SRB) (Sigma, Darmstadt, Germany) at a concentration of 0.4% in 1% acetic acid. The plates were washed five times with 1% acetic acid and then dried again for 1 h at 60 °C. SRB was dissolved in 10 mM Tris pH 10.5 and the extinction of the stained supernatant was measured photometrically at 546 nm.

### 4.5. TUNEL Staining

TdT-mediated dUTP nick end labeling (TUNEL) staining was performed with an in-situ cell death detection kit purchased from Roche (Sigma Aldrich, Munich, Germany) as recommended by the manufacturer. In brief, RAW264.7 cells were incubated for 24 h with TSA and SAHA. Then, cells were rinsed with PBS and fixated with 4% paraformaldehyde. Blocking was performed with 3% H_2_O_2_ and lysis with 0.1% triton X-100. Afterwards, TUNEL labeling started for 60 min with labeling solution and then 30 min with POD converter. As peroxidase substrate, 3, 3 -diaminobenzidine (DAB) was added for 5 min, the slides were rinsed with PBS for 3 times and then the staining was analyzed by microscopy (Zeiss Primovert, Jena, Germany).

### 4.6. Polymerase Chain Reactions (PCR)

Total RNA was extracted from RAW264.7 cells as described previously (Chomczynski, 1993). Two-hundred nanograms of total RNA was used for the reverse transcription (RT), which was performed with the Thermo Scientific Verso cDNA system (Thermo Fisher Scientific, Schwerte, Germany). Twenty nanograms RNA equivalent were subjected to quantitative real-time PCR (qRT-PCR) in a QuantStudio 5 Real-Time PCR system (Thermo Fisher Scientific, Germany) using a TaqMan™ Fast Advanced Master Mix (Thermo Fisher Scientific, Germany) with SYBR Green fluorescence staining. Expression of PCR products was determined and normalized to glyceraldehyde-3-phosphate dehydrogenase (GAPDH) mRNA. The following gene-specific mouse primers were used:
COX-2FW 5’-AGACACTCAGGTAGACATGATCTACCCT-3′
RV 5′-GGCACCAGACCAAAGACTTCC-3′IKKεFW 5′-GTACAAGGCCCGAAACAAGA-3′
RV 5′-TCCTCCACTGCGAATAGCTT-3′IL-1βFW 5′-CTGGTGTGTGACGTTCCCATTA-3′
RV 5′-CCGACAGCACGAGGCTTT-3′IL-10FW 5′-GCTCTTACTGACTGGCATGAG-3′
RV 5′-CGCAGCTCTAGGAGCATGTG-3′iNOSFW 5′-ACACAGCGCTACAACATCCT-3′
RV 5′-TGGAGCACAGCCACATTGAT-3′TNFαFW 5′-GCTGAGCTCAAACCCTGGTA-3′
RV 5′-CGGACTCCGCAAAGTCTAAG-3′GAPDH FW 5′-CAATGTGTCCGTCGTGGATCT-3′
RV 5′-GTCCTCAGTGTAGCCCAAGATG-3′

The cycle number at which the fluorescence signals cross a defined threshold (Ct-value) is proportional to the number of RNA copies present at the start of the PCR. The threshold cycle number for the specific mRNA was standardized by subtracting the mean Ct-value of GAPDH from the Ct-value of the specific PCR product of the same sample.

### 4.7. ChIP Assay

Chromatin immunoprecipitation was performed using a commercially available SimpleChIP Plus Assay (Cell Signaling, Heidelberg, Germany) as recommended by the manufacturer. Briefly, RAW 264.7 cells were seeded in 15 cm cell culture plates at a density of 5 × 10^6^. After attachment of the cells, incubation with TSA and SAHA took place for 16 h. Then, cells were harvested, DNA-protein cross-linking was performed with 37% formaldehyde and nuclei were prepared. After chromatin digestion and purification with RNase A, the DNA concentration was determined. Five microgram chromatin preparation was immunoprecipitated with protein G agarose beads and antibodies against acetyl-histone H3 and histone H3. As negative control, normal rabbit IgG was used. Subsequently, chromatin was eluted from the antibodies and cross-links were reversed, DNA was purified and subjected to PCR with primers against IKKε promoter and the positive control (RLP30).

The following primers were used for amplification of the IKKε promoter:

FW 5′-GATCGACAAGTGCAATCCCC-3′
RV 5′-AGCCACTCAAATGCACTGTG-3′

### 4.8. Western Blot Analysis

For the preparation of crude protein extracts, cells were treated as indicated and then harvested by a rubber policeman. Cell lysis was performed in PhosphoSafe Extraction Buffer (Merck, Darmstadt, Germany) containing protease inhibitor (1 mM Pefabloc) (Alexis Biochemicals, Lausen, Switzerland). To remove cellular debris, extracts were centrifuged at 16,800× *g* for 30 min at 4 °C. The supernatants were stored at –80 °C.

For the preparation of cytosolic and nuclear fractions, cells were harvested in PBS and centrifuged for 1 min at 10,000 rpm. After resuspension of the pellets in 1 mL lysis buffer I (10 mM Tris-HCl, pH 7.4, 10 mM NaCl, 3 mM MgCl_2_, 1 mM PMSF, 2 mM DTT), cell lysis was performed by 10 min incubation on ice with subsequent addition of Nonidet-40 (NP-40) (final concentration 0.5%) and centrifugation at 400× *g* for 5 min. Cytosolic extracts in the supernatant were collected and frozen at −80 °C until further analysis. The nuclear fraction in the pellet was resuspended with lysis buffer I, centrifuged again and then the nuclei were lysed in 2 vol. lysis buffer II (20 mM HEPES-KOH, pH 7.4, 600 mM KCl, 0.2 mM EDTA, 1 mM PMSF, 2 mM DTT) for 30 min on ice. After centrifugation for 10 min at 10,000× *g*, the supernatant was collected and 1 vol. lysis buffer III (20 mM Hepes-KOH, pH 7.4, 0.2 mM EDTA, 0.5 mM PMSF, 2 mM DTT) as well as glycerol (final concentration 20%) was added. Nuclear extracts were stored at −80 °C until further analysis.

Protein lysates (crude extracts and cytosolic fraction 30 µg, nuclear fraction 5 µg) were subjected to 8%, 10% or 12% SDS-PAGE. After separation, proteins were transferred onto nitrocellulose membranes by wet-blotting. Subsequently, membranes were stained with Ponceau red solution to confirm equal loading and then unspecific binding sites were blocked for 60 min at room temperature in Odyssey blocking reagent (1:2 in 0.1 M PBS, pH 7.4) (Licor, Bad Homburg, Germany). Hybridization with primary antibodies against IKKε, p65, I-κBα (all 1:250, Cell Signaling Technology, Heidelberg, Germany), COX-2 (1:1000, Santa Cruz, Heidelberg, Germany) and iNOS (1:200 Sigma-Aldrich, Germany) was performed overnight at 4 °C in blocking buffer. Then, the blots were washed three times in PBS with 0.1% Tween 20 and incubated with an IRDye 800- or IRDye 700-conjugated secondary antibody (Licor, Bad Homburg, Germany, 1:5000 in blocking buffer) for 60 min at room temperature. After final washing, the protein-antibody complexes were scanned in an Odyssey Infrared Imaging System (Licor, Bad Homburg, Germany). Beta-Actin (1:1000 Sigma-Aldrich, Deisenhofen, Germany) served as loading control for total and cytosolic extracts and PCNA (1:1000, Santa Cruz, Heidelberg, Germany) as loading control for nuclear extracts, respectively. Protein-specific bands on the blots were analyzed densitometrically using Image Studio Lite Software (Licor, Bad Homburg, Germany).

### 4.9. p65 TransAM ELISA

Nuclear extracts were prepared using a nuclear extract kit (Active Motif, La Hulpe, Belgium) according to the manufacturer’s instructions. Then these nuclear extracts were subjected to TransAM NF-κB p65 transcription factor ELISAs according to the manufacturer’s instructions (Active Motif, La Hulpe, Belgium). Briefly, 10 µg nuclear protein were allowed to bind to an oligonucleotide-coated plate. NF-κB-p65 was detected by incubation with specific primary antibodies and an HRP-conjugated secondary antibody. The colorimetric read-out was measured photometrically and is proportional to transcription factor activity.

### 4.10. Flow Cytometry

Cell cycle distribution and apoptosis induction were evaluated by flow cytometry using propidium iodide (PI, Sigma, Darmstadt, Germany) staining on a flow cytometer (FACSCanto II, Becton Dickinson, Heidelberg, Germany) as described previously (Moser et al., 2016). In brief, RAW264.7 cells were incubated with 5 and 10 µM SAHA, 100 and 500 nM TSA or DMSO for 4 and 24 h, respectively. At the end of the incubation period, cells were scraped by a rubber policeman. After two washing steps with PBS, cells were fixed for at least 12 h with 80% ethanol (*v/v*) at −20 °C, then washed again with PBS before incubation with 0.125% Triton X- 100 for 5 min on ice. Afterwards, cells were washed again with PBS and then stained with propidium iodide (20 µg/mL) (Sigma, Darmstadt, Germany) in PBS containing 0.2 mg/mL RNase A (Qiagen, Hilden, Germany). 100,000 cells/samples were counted in the flow cytometer and subG1, G1, S and G2/M fractions were quantified using FlowJo software and manual gating.

### 4.11. Data Analysis

Statistical evaluation has been done with GraphPad Prism 7.03 for Windows. Data are presented as mean ± S.E.M. Data were either compared by univariate analysis of variance (ANOVA) with subsequent Dunnett´s multiple comparisons test or by Student’s *t*-test. For all tests, a probability value *p* < 0.05 was considered as statistically significant. 

## Supplementary Materials

Supplementary materials can be found at https://www.mdpi.com/1422-0067/21/23/8943/s1.

## Abbreviations

AcH3	Acetyl histone H3
AmD	Actinomycin D
BMM	Bone marrow macrophages
ChIP	Chromatin immunoprecipitation
COX-2	Cyclooxygenase 2
DMSO	Dimethylsulfoxide
ELISA	Enzyme linked immunosorbent assay
FACS	Fluorescence activated cell sorting
FCS	Fetal calf serum
GAPDH	Glyceraldehyde-3-phosphate dehydrogenase
HAT	Histone acetylase
HDAC	Histone deacetylase
HDACi	Histone deacetylase inhibitor
IFN	Interferone
IκBa	Inhibitor kappa B protein alpha
IKKε	I-κB kinase epsilon
IL	Interleukin
LPS	lipopolysaccharide
M-CSF	murine macrophage colony stimulating factor
iNOS	Inducible nitric oxide synthase
NFκB	Nuclear factor kappa B
PCNA	Proliferating-Cell-Nuclear-Antigen
RLP30	Receptor-like protein 30
qRT-PCR	Quantitative reverse transcription polymerase chain reaction
TCA	Trichloroacetic acid
TLR	Toll-like receptor
TNF	Tumor necrosis factor
TSA	Trichostatin A
TUNEL	TdT-mediated dUTP nick end labeling
SAHA	Suberoylanilide hydroxamic acid
SRB	Sulforhodamine B
