# Supplementary material for: Inhibition of HDAC Enzymes Contributes to Differential Expression of Pro-Inflammatory Proteins in the TLR-4 Signaling Cascade
Source: Int J Mol Sci. 2020 Nov 25;21(23):8943. doi: 10.3390/ijms21238943 (PMC7728096; doi:10.3390/ijms21238943)
Supplement: Supplementary file 1 [file ijms-21-08943-s001.pdf]

Suppl. Figure 1: Regulation of iNOS and TNFα mRNA in IKKε wildtype and knock-out bone marrow derived macrophages

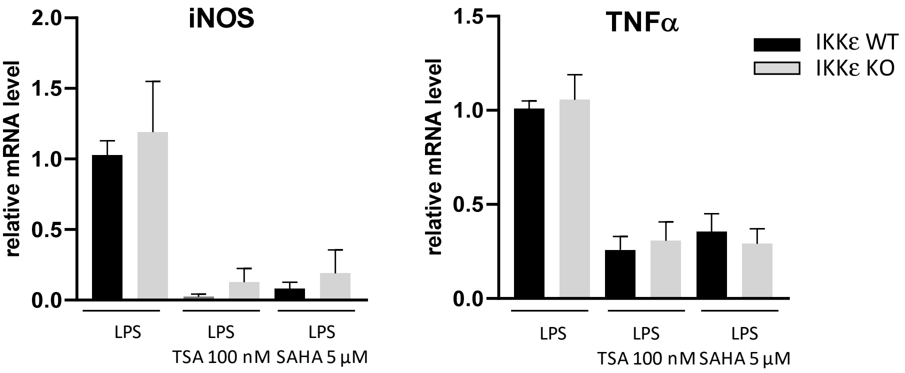

Suppl. Figure 2: Evaluation of HDAC siRNA

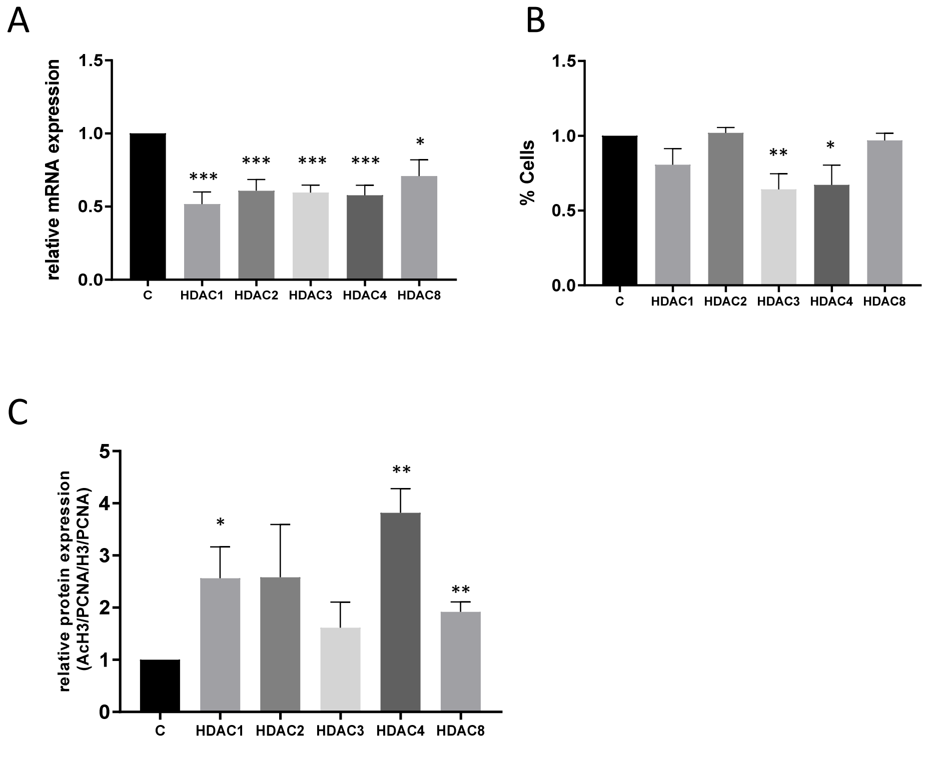

Figure Legend

Suppl. Fig. 1: *Regulation of iNOS and TNFα mRNA in IKKε wildtype and knock-out bone marrow-derived macrophages*

qRT-PCR analysis of iNOS and TNFα mRNA expression in bone marrow-derived macrophages from IKKε wildtype and knock-out mice (n=3). For better comparison, expression of all genes was normalized to LPS-treated control cells which have been set as "1".

Suppl. Fig. 2: *Evaluation of HDAC siRNA*

(A) qRT-PCR showing the regulation of different HDACs mRNAs after transfecting cells with the respective HDAC siRNA. Vehicle-treated cells served as control (n=9). (B) WST cell proliferation assay 48 h after cells transfection with the respective siRNAs (n=5). The proliferation rate of vehicle-treated cells was set as "1". (C) Western Blot analysis of nuclear histone H3 acetylation 48 h after transfection of cells with HDAC siRNA. The diagram shows the densitometric analysis of all Blots (n=3). For better comparison, expression of all genes was normalized to vehicle-treated control cells which have been set as "1". *$p<0.05$, **$p<0.01$, ***$p<0.001$, statistically significant difference as compared to vehicle-treated control.
